# Symptoms and Risk of Obstructive Sleep Apnea in Patients Attending Primary Health Care Clinics in Riyadh, Saudi Arabia

**DOI:** 10.7759/cureus.33543

**Published:** 2023-01-09

**Authors:** Bader AlTulaihi, Maha T Alassafi, Fuoz H Alanazi, Linah N Ban Owaiwid, Hind Alasmari, Malak M Alhaddab, Maram Albalawi

**Affiliations:** 1 Family Medicine, King Abdulaziz Medical City Riyadh, Riyadh, SAU; 2 Family Medicine, Ministry of National Guard - Health Affairs, Riyadh, SAU; 3 College of Medicine, King Saud bin Abdulaziz University for Health Sciences, Riyadh, SAU; 4 College of Medicine, King Saud Bin Abdulaziz University for Health Sciences, Riyadh, SAU; 5 Biostatistics, King Abdullah International Medical Research Center, Riyadh, SAU

**Keywords:** risk factors for osa, berlin questionnaire, s: prevalence, primary health care centers, kingdom of saudi arabia (ksa), obstructive sleep apnea (osa)

## Abstract

Background

Obstructive sleep apnea (OSA) is a breathing disorder that occurs while sleeping, characterized by periods of partial or complete airway collapse associated with a drop in oxygen saturation or arousal from sleep. OSA is associated with significant health consequences, including hypertension, obesity, coronary artery disease, metabolic dysfunction, cognitive decline, and cancer progression.

Methods

A cross-sectional study was conducted at the four primary healthcare centers in the Ministry of National Guards Health Affairs (MNGHA) in Riyadh, Saudi Arabia. The study included every adult 18- 65 years old. After informed consent was obtained, interviews were conducted using the Berlin questionnaire (BQ) to assess participants’ prevalence and risk (OSA). SAS software version 9.4 was used to enter and analyze the data.

Results

A total of 400 primary care attendants were approached. The overall prevalence of high-risk apnea was 21%. There was a significant difference in the high risk of apnea between a BMI greater than 30 and a BMI lower than 30 (p< 0.0001). There was a significant difference in the high risk of apnea between hypertension and normal blood pressure (p< 0.0001).

Conclusion

The BQ assessed the predictive factors related to the development and prevalence of OSA and showed a predominance in those with a BMI over 30 Kg/m^2^ or with a previous diagnosis of hypertension with no significant related risk among gender and age factors.

## Introduction

Obstructive sleep apnea (OSA) is a breathing disorder that occurs while sleeping, characterized by periods of a partial or complete airway collapse associated with a drop in oxygen saturation or arousal from sleep [1]. This disorder resulted in non-restful sleep, loud snoring, and observed apneas [2]. Moreover, daytime sleepiness and morning headaches are other symptoms [2]. OSA is associated with significant health consequences, including hypertension, obesity, coronary artery disease, metabolic dysfunction, cognitive decline, and cancer progression [3,4]. Sleep-related accidents are also strongly associated with OSA [5].

A study done in the United States, Germany, and Spain included 6,223 participants who assessed the symptoms and risk of sleep apnea in primary care. The United States reported a higher OSA risk score than Germany and Spain for both men and women. Overall, the study confirmed the high rate of patients with behaviors resulting in individuals, and societal risks, such as frequent sleepiness and drowsy driving [6]. Five hundred twenty-seven adults were evaluated for symptoms and risk of obstructive sleep apnea in Kermanshah, Iran, using the Berlin questionnaire, which showed 27.3% of the participants were classified as high risk for OSA. In summary, more than 1 in 4 individuals in Kermanshah presented with symptoms and risk factors for OSA [7]. In a cross-sectional study of 1,205 patients, symptoms and risk of obstructive sleep apnea were evaluated. Based on the berlin questionnaire, 16.8% of the patients present with a high risk for obstructive sleep apnea. Moreover, the group with the increased risk is related to the male gender, obesity, tobacco smoking, hypertension, nasal congestion, and age ≥30 years. Also, high-risk participants were less likely to keep a job [8]. Being at high risk for OSA was found to be among 48% of participants with (a BMI≥30 kg/m^2^) compared to 2% of the participants with a BMI<20 kg/m^2^. A similar pattern was seen in a study conducted in America in 2005 with 1,506 adults; 59% of the obese participants were at high risk for OSA, while 2% of the participants with (a BMI<20 kg/m^2^) were at increased risk [8,9]. In the Middle East, the high prevalence of obesity, smoking, and diabetes play a significant role in the risk factors for OSA [10]. The majority of OSA-related obesity is increasing rapidly in the Middle East region (Saudi Arabia, United Arab Emirates, Qatar, Bahrain, Kuwait, and Oman) [11]. Limited studies in Jordan, Saudi Arabia, and the United Arab Emirates showed that the risk of OSA evaluated from symptoms reported by patients attending primary health care clinics was very high in UAE adult citizens [12]. Specifically, a study conducted in Dubai showed that 20.9% of patients who attended primary care were at increased risk for OSA (22.9% males, 19.5% females) [11]. In Jordan, it ranged from 16.8% [8] while 33.0% in Saudi Arabia [13]. A study in Saudi Arabia reported that one in 3 middle-aged Saudi males is at risk for OSA in primary care [14]. Another study in Saudi Arabia showed that the rate and risk factors of OSA syndrome in the Saudi population are similar to those observed in Western studies [15]. Moreover, 52.4% is the high risk of OSA in Saudi Arabia, and women are more likely to be at increased risk of OSA than men [16].

Many factors, including age, gender, BMI, and hypertension, were established to be associated with the prevalence of obstructive sleep apnea [15]. Increasing literature is highly important to have an update on the majority of OSA among people attending primary healthcare clinics in Saudi Arabia. As most current studies target the elderly and male gender, this study compares the prevalence of OSA among males and females and includes young adults and the elderly. 

The main aim of this study was to estimate the prevalence and risk factors of obstructive sleep apnea among patients attending primary health care centers. Moreover, this study was also intended to identify the association between specific risk factors like obesity, gender, age, hypertension, and the development of Obstructive Sleep Apnea (OSA).

## Materials and methods

A cross-sectional study was conducted at the four primary health care centers (PHC) (King Abdulaziz Housing clinics (Iskan), Dirab clinic, National Guard Comprehensive Specialized Centre (NGCSC), and Khashmalan clinic) affiliated with the Ministry of National Guards Health Affairs (MNGHA) in Riyadh, Saudi Arabia. In MNHGA’s primary health care centers, which are distributed across Saudi Arabia, all medical services are provided under the supervision of family medicine specialties.

All four PHC centers are delivered through King Abdulaziz Medical City (KAMC), providing primary curative and preventive health services. Each center includes walk-in and appointment booking services with a minor emergency room setting.

 After obtaining informed consent, patients were selected and interviewed in each primary health care center in KAMC. Every Adult patient from 18 to 65 years old attending the adult health clinic of the four PHCCs ( Khashm al ‘An, Iskan, Om Alhamam, Dirab) was included in the study with no exclusion criteria.

To calculate the sample size, the Openepi website was used. The population of KAMC-PHC, as per the latest survey, was 420,000 in 2017, with a confidence level of 95% as convention and a 5% of error margin; the sample size was calculated to be 384 subjects. To compensate for any loss of participants, the sample was considered to be 400. Using a non-probability convenient sampling technique, the patients were interviewed by trained undergraduate medical students in their 5th and 6th year through an online survey.

Berlin questionnaire (BQ) was used to assess the prevalence and risk of Obstructive Sleep Apnea (OSA) among all participants. BQ is a validated tool for self-report screening of OSA developed in 1996 [17]. BQ has a sensitivity of 0.86, a specificity of 0.77, and a positive predictive value of 0.89 for a high-risk group, as predicted by the questionnaire [17]. Patients were classified into high-risk or low-risk based on their responses to the individual items and their overall scores in the symptom categories. High-risk patients scored two positive categories or more, and low risk if one or no category scored positive [17]. The questionnaire has three categories with a total of ten questions. The first category consists of five questions about snoring and loudness, frequency and if it bothers other people, and frequency of observed apnea. The second category concerns fatigue, daytime sleepiness, and drowsy driving. The first two categories were considered positive if they scored two or more [17]. The third category addresses the presence of hypertension, and it was scored positive for patients with hypertension or Body Mass Index (BMI) of more than 30 kg/m^2^ [17].

SAS software version 9.4 (SAS Institute Inc., Cary, North Carolina, USA) was used to enter and analyze the data. Descriptive statistics were used to introduce participants’ characteristics and the answers to the different questionnaires. Demographic characteristics were presented as frequency and percentage. For continuous variables, data are presented as (n, n missing, mean, standard deviation, median, quartile range, minimum and maximum). The association between having OSA and gender, BMI, and hypertension was analyzed using the Chi-square test. Results are reported as percent and 95% CI. The risk factors of high apnea were analyzed using logistic regression. The dependent variable was apnea (high risk/low risk), and the independent variables included gender, BMI, and hypertension. Results were reported as odds ratios, 95% confidence intervals, and corresponding p-values. A p-value less than 0.05 was declared as significant.

King Abdullah International Medical Research Center (KAIMRC) approved the study protocol and questionnaire. Participants’ privacy and confidentiality were assured and respected, no identifiers were collected, and all hard and soft copies of data were stored within MNGHA premises and accessed by the research team only.

## Results

During the study period of 3 months, a total of 400 primary care attendants were approached. The mean age of the patients was 37.07±12.50 (range of 18-65). A total of 254 (63.5%) were female. Their mean BMI was 28.23±6.00 kg/m^2^, 67.25% of participants have BMI less than 30, and 82.5% do not have hypertension.

HR-OSA was present in 84 (21.00%) patients. Positive response in category 1 was present in 112 (28.00%), 42 (10.75%) in category 2, and 157 (39.25%) in category 3. The contribution of each item in the questionnaire to the OSA risk is described in table 1.

**Table 1:** Response to obstructive sleep apnea questionnaire N=400. a Positive response as defined in the methodology section b Obesity: BMI≥30 kg/m2

Questionnaire items	Frequency of positive response ^a ^N(%)
Category 1	112 (28.00)
Presence of snoring	155 (38.75)
Louder than talking	31 (20.00)
Snoring: ≥3–4 times per week	80 (51.95)
Snoring bothers other people	76 (49.35)
Witnessed apnea during sleep: ≥3-4 times per week	20 (13.07)
Category 2	43 (10.75)
Feeling tired or fatigued after sleep: ≥3–4 times per week	115 (28.75)
Sleep while driving a vehicle	81 (20.25)
Sleeping while driving: ≥3–4 times per week	29 (36.25)
Category 3	157 (39.25)
Hypertension	70 (17.50)
Obesity ^b^	131 (32.75)

The prevalence of high-risk apnea was 21% (95% CI 16.99, 25). The prevalence of high-risk apnea in males was 8.5% (95% CI 5.75, 11.24), while in females was 12.5% (95% CI 9.24, 15.75). There was no significant difference in the high risk of apnea between males and females. The prevalence of increased risk of apnea in patients with a BMI greater than 30 was 16% (95% CI 12.39, 19.6), while in patients with BMI lower than 30 was 5% (95% CI 2.85, 7.14). There was a significant difference in the high risk of apnea between a BMI greater than 30 and a BMI lower than 30 (p< 0.0001). The prevalence of high-risk apnea in patients with hypertension was 11.25% (95% CI 8.14, 14.35), while in patients with normal blood pressure was 9.75% (95% CI 6.83, 12.66). There was a significant difference in the high risk of apnea between hypertension and normal blood pressure (p< 0.0001) (table 2).

**Table 2:** Obstructive sleep apnea risk according to socio-demographic characteristics and relevant associated factors N= 400. a: HR-OSA for high risk for obstructive sleep apnea (n= 84) 21.00%, b: LR-OSA for low risk for obstructive sleep apnea (n = 316) 79.00%, c: Obesity: BMI ≥ 30 kg/m2

Demographic Variables	HR-OSA^a^ n (%)	LR-OSA^b ^n(%)	P value	(mean ±SD)
Age				37.07±12.50
Weight				74.03±15.96
Height				162.37±8.46
BMI				28.10±5.88
Gender			0.4851	
Male	34 (8.50)	112 (28.0)		
Female	50 (12.0)	204 (51.0)		
Obesity ^c^			<0.0001	
Yes	64 (16.0)	67 (16.75)		
No	20 (5.0)	249 (62.25)		
Hypertension			<0.0001	
Yes	45 (11.25)	25(6.25)		
No	39 (9.75)	291 (72.75)		

**Figure 1:**
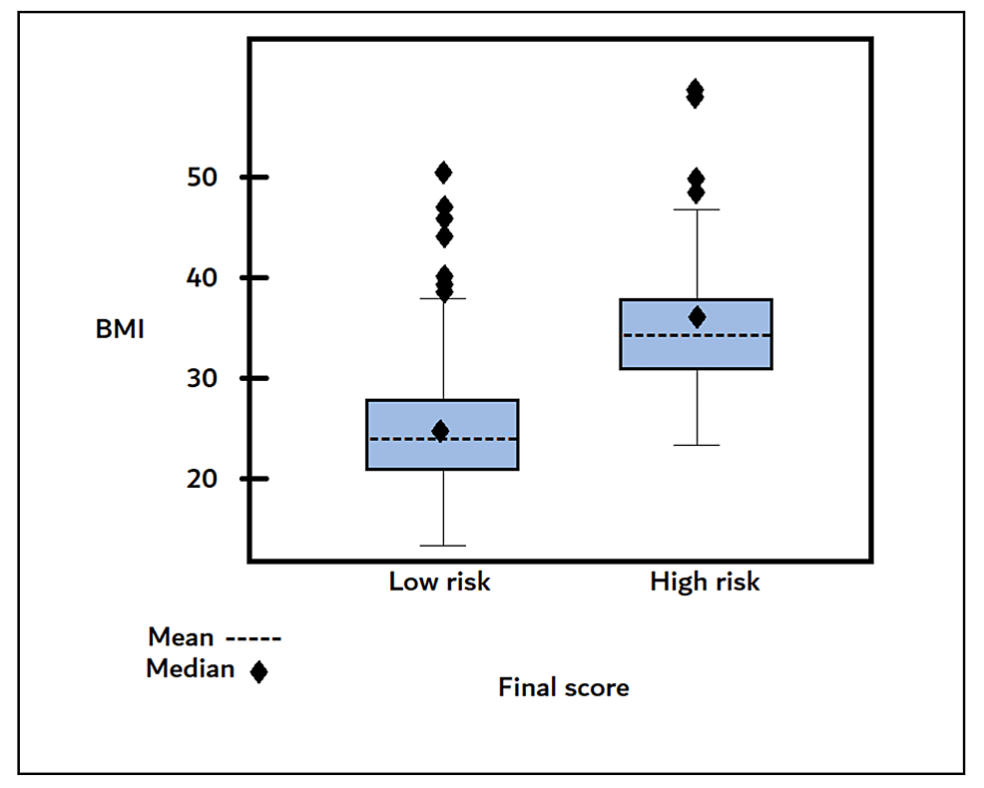
Final score with BMI; The subjects with high BMI are more likely to be at higher risk.

**Figure 2:**
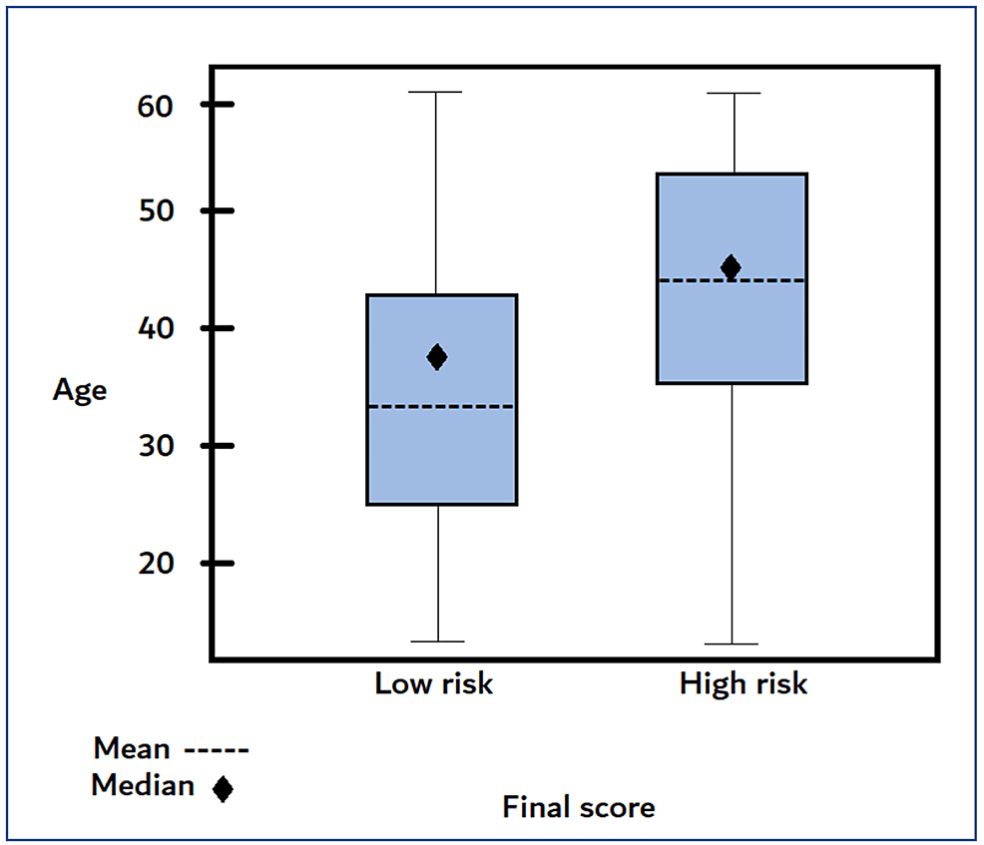
Final score with age. The subjects with advanced age are more likely to be at higher risk.

Among the explored factors, hypertension was a significant predictor that was 10.187 times more likely to develop apnea than normal blood pressure (OR 10.187, 95% CI 5.19, 19.97, p<0.0001) (table 3). Also, BMI greater than 30 was a significant predictor; 9.521 times more likely to have a high risk of apnea than patients with a BMI lower than 30 (OR 9.521, 95% CI 5.10, 17.76, p<.0001) (table 3, figure 1). In addition to age, subjects older than 45 were more likely to be at higher risk (figure 2).

**Table 3:** Logistic regression model demonstrating the relationship between high blood purser, BMI, Gender, and the final score.

Independent Variable	Adjusted Odd Ratio (AOR)	95% CI for AOR Lower	95% CI for AOR Upper	p-Value
Do you have high blood purser (Yes vs. No)	10.187	5.195	19.977	
BMI greater than 30 (Yes vs. No)	9.521	5.103	17.765	
Gender (Male vs. Female)	1.249	0.669	2.334	0.4851

## Discussion

Our study addresses the risk factor and prevalence of HR-OSA and OSA symptoms in primary care in KSA. In this study, the HR-OSA was present in 84 (21%) patients, which is less than the previously reported studies in Saudi Arabia (29.8%); however, the previous study assessed sleep apnea in Saudi Arabia using both the BQ and the ESS [18]. Moreover, there are older studies done to assess the prevalence of symptoms and risks of obstructive sleep apnea in middle-aged Saudis using BQ that showed that (33.3%) of men and (39%) of women were considered at high risk for sleep apnea [13,14]. In our study, The prevalence of high-risk apnea in males was (8.5%), while in females was (12.5%).

To our extensive research, this is the first Saudi study conducted on a younger age group. In this study, the mean age of the interviewed patients was 37.07±12.50 (range of 18-65), which is younger than all previously reported studies in KSA. Thus, this may be the reason for the lower result in our study. Despite the previously reported Saudi studies, we found no statistically significant difference in the high risk of apnea between genders [13,14,18]. In Europe, there was a wide range between studied regions (19.9%- 66.7%) and 26.3% (range 23.2%-44.8%) [6].

In the current study, positive response in category one was present at (28.00%) which is less than the reported studies in both Europe (43.5%) and the USA (43.3%), [6] yet it was higher than the reported in Jorden (18.8%) [8]. Similarly, the positive response in category three was (39.25%) which is higher than reported in Jordan (29.2%) and lower than that in the USA (44.8%) and Europe (37.1%). On the other hand, (10.75%) of positive responses in category two, which is much lower than reported studies in Jordan (20.3%) USA (32.4%) and comparable to Europe (11.8%). The reason behind the decreased rates of OSA symptoms, snoring, and the younger age of the participants could explain daytime sleepiness in this study and Jordin. However, our mean age was higher than the one reported in the Jordanian study (the mean age was 32.2 years).

In a population-based study in the united states, 59% of individuals with BMI> 30KG/M had a high risk of OSA, compared with only 0.2% of individuals with BMI<20kg/m2 had a high risk of OSA [9]. In another study conducted in Saudi Arabia, a total of 319 patients attending family medicine clinics were screened for the risk of sleep apnea. Those classified as obese compared to those not obese had 5.7 times the odds of being classified as high risk for sleep apnea [18]. All These results from both studies showed that obesity is a strong predictor for OSA risk. A similar pattern was seen in this study; the prevalence of high risk of apnea in patients with a BMI greater than 30 was 16% (95% CI 12.39, 19.6), while in patients with BMI, lower than 30 was 5% (95% CI 2.85, 7.14).

According to various research, patients with hypertension were found to have an increased risk of developing sleep apnea. The primary care-based study in Jordan estimated the prevalence of symptoms and risks of OSA using the Berlin questionnaire. The results showed that in high-risk patients for obstructive sleep apnea, 62.0% have hypertension [8]. Ahmad et al. studied the risk of sleep apnea in a family medicine clinic and found that compared to those with no hypertension, those with hypertension had 4 times the odds of being classified as high risk for sleep apnea [18]. Also, the Systemic review and meta-analysis included twenty studies assessing the relationship between OSA and hypertension. In 16 studies, the OSA was classified into three grades based on an apnea-hypopnea index; the more the score of AHI, the more severe the OSA. Moreover, the results showed that the more risk the OSA, the higher the risk of hypertension [19]. In our study, the prevalence of high risk of apnea in patients with hypertension was 11.25%, while in patients with normal blood pressure was 9.75%.

In the current study, some limitations need to be addressed. The study was done in primary care, which may only be representative of some populations. A respectable number of primary care patients may have other comorbidities, which make them at higher risk of OSA. Moreover, the results may overestimate the actual burden of the disease in the general population.

## Conclusions

In conclusion, updated data for the prevalence and risk of OSA among the Saudi population was needed to help encourage primary healthcare practitioners to early detection of the disease or assess the risk factors present in some patients. The BQ assessed the predictive factors related to the development of OSA in adults between 18 to 65, which showed a predominance in those with BMI over 30 Kg/m^2^ or those with hypertension. However, there was no significant related risk between gender and age. Further efforts should be made to raise awareness of the disease, mainly by primary healthcare professionals, to help to prevent the disease complications and control the risk factors.
